# Inulin Supplementation Lowered the Metabolic Defects of Prolonged Exposure to Chlorpyrifos from Gestation to Young Adult Stage in Offspring Rats

**DOI:** 10.1371/journal.pone.0164614

**Published:** 2016-10-19

**Authors:** Julie Reygner, Lydia Lichtenberger, Ghada Elmhiri, Samir Dou, Narges Bahi-Jaber, Larbi Rhazi, Flore Depeint, Veronique Bach, Hafida Khorsi-Cauet, Latifa Abdennebi-Najar

**Affiliations:** 1 UP-EGEAL 2012.10.101, Institut Polytechnique LaSalle Beauvais, Beauvais, France; 2 Laboratoire Périnatalité et Risques Toxiques (PERITOX), UMR-I01 INERIS, Jules Verne University of Picardy, Amiens, France; Wageningen University, NETHERLANDS

## Abstract

Increasing evidence indicates that chlorpyrifos (CPF), an organophosphorus insecticide, is involved in metabolic disorders. We assess the hypothesis whether supplementation with prebiotics from gestation to adulthood, through a modulation of microbiota composition and fermentative activity, alleviates CPF induced metabolic disorders of 60 days old offspring. 5 groups of Wistar rats, from gestation until weaning, received two doses of CPF pesticide: 1 mg/kg/day (CPF1) or 3.5 mg/kg/day (CPF3.5) with free access to inulin (10g/L in drinking water). Then male pups received the same treatment as dams. Metabolic profile, leptin sensitivity, insulin receptor (IR) expression in liver, gut microbiota composition and short chain fatty acid composition (SCFAs) in the colon, were analyzed at postnatal day 60 in the offspring (PND 60). CPF3.5 increased offspring’s birth body weight (BW) but decreased BW at PND60. Inulin supplementation restored the BW at PND 60 to control levels. Hyperinsulinemia and decrease in insulin receptor β in liver were seen in CPF1 exposed rats. In contrast, hyperglycemia and decrease in insulin level were found in CPF3.5 rats. Inulin restored the levels of some metabolic parameters in CPF groups to ranges comparable with the controls. The total bacterial population, short chain fatty acid (SCFA) production and butyrate levels were enhanced in CPF groups receiving inulin. Our data indicate that developmental exposure to CPF interferes with metabolism with dose related effects evident at adulthood. By modulating microbiota population and fermentative activity, inulin corrected adult metabolic disorders of rats exposed to CPF during development. Prebiotics supply may be thus considered as a novel nutritional strategy to counteract insulin resistance and diabetes induced by a continuous pesticide exposure.

## Introduction

During the last decade, the incidence of obesity and diabetes has dramatically increased all over the world. According to the World Health Organization (WHO), the world prevalence of diabetes estimated to be at 2.8% in 2000 will reach 4.4% in 2030 [1]. Western diet and lack of physical exercise are currently related to this burden of metabolic diseases. Recently, epidemiological and animal studies pointed out the involvement of chemical exposure during pregnancy and lactation in the increasing incidence of this metabolic syndrome [2–5].

Although it is increasingly restricted to the US and Europe, the highly-lipophilic organophosphorus (OP) compound Chlorpyrifos (CPF) is one of the most frequently used non-persistent insecticides worldwide and is commonly found in fruits and vegetables [6]. Numerous studies at high level exposure have endorsed the neurotoxic effects of CPF in both human and animal models [7]. CPF exerts its systemic toxicity by irreversibly inhibiting acetylcholinesterase [8]. At low-levels this compound targets cell signaling cascades that govern neuronal and hormonal signals, which are linked to homeostatic balance and cellular differentiation.

Recent epidemiological studies and investigations in experimental animal models support the effect of early exposure of CPF in the ontogeny of diabetes [6,9,10]. Neonatal exposure to CPF displays hyperinsulinemia and hyperlipidemia in adulthood rat, two major risk factors for type 2 diabetes mellitus (T2D) and atherosclerosis. These findings extend Barker’s hypothesis [11] showing that in the absence of intrauterine growth restriction, CPF during early development can result in permanent changes in the physiology and metabolism resulting in increased metabolic risks in adulthood.

Gut microbiota exerts a significant role in the pathogenesis of the metabolic syndrome as confirmed by studies conducted both in human and animal models [12–14]. In fact, gut microbiota plays a great variety of functional roles impacting human physiology. It modulates host nutrition by the production of vitamins and fermentation of food components indigestible by the host, protects against pathogens [15] and drug metabolism and influences intestinal epithelial homeostasis [16]. An impairment of the fine balance between gut microbes and host’s interactions induces the intestinal translocation of bacterial fragments and the development of “metabolic endotoxemia”, leading to systemic inflammation and insulin resistance [17]. Clinical and animal studies on obesity and T2D showed a shift in the pattern of the gut microbiota, in particular a decrease in the ratio of *Firmicutes/Bacteroidetes* for obesity [14] and a lower proportion of Clostridiales for T2D [18]. Compelling evidence suggests that oral supplementation with selectively fermented oligosaccharides (known as prebiotics) improves these metabolic disorders via several mechanisms [19–21]. Moreover, prebiotics are likely associated with the increase in Bifidobacteria and Lactobacilli and the production of short chain fatty acids (SCFAs), which are involved in the modulation of the host metabolism [22]. For example, feeding genetically or diet-induced obese mice with prebiotics significantly increased the abundance of *Akkermansia muciniphila*, which was correlated with an improved metabolic status [21,23]. Other studies have shown that prebiotics reinforce the gut barrier, increase satiety by promoting gut hormones, improve glucose tolerance, counteract hepatic steatosis (lipogenesis) and insulin resistance [24].

We recently showed that neonatal exposure to CPF disturbed the microbiota composition specifically the proportion of Lactobacilli in the colon at PND 60 [25]. In the current study, we wanted to test whether early prebiotic supplementation counteracts the metabolic disorders induced by early exposure to CPF. Rats were exposed from pregnancy to weaning, to two doses of CPF (1mg/Kg/day, CPF1) and (3.5 mg/Kg/day, CPF3.5), either alone or in association with inulin. Inulin was administered along with drinking water at a dose of 10g/L. We intended to induce CPF exposure from gestation to weaning to mimic the effect of CPF exposure in a real human neonate during its development. We evaluated the effect of the different treatments in rats on lipid and glucose metabolism, insulin and leptin, gut microbiota composition and SCFA production at 60 days of age. The supplementation with inulin is relevant in this study because it may help to identify new properties of prebiotics in both the mother and the child exposed to pesticides, and to develop new strategies against metabolic programming in later life.

## Materials and Methods

### Chemicals

Chlorpyrifos (O, O-diethyl, O-(3,5,6-trichloro-2-pyridyl) phosphorothioate), purity 99.8% ± 0.1%, was supplied by LGC Standards (Molsheim, France). It was dissolved in rapeseed oil (MP Biomedicals, Illkirch, France), which served as a vehicle and administrated daily by gavage at a dose of 1 mg/kg of BW/day (CPF1) or 3.5 mg/kg of BW/day (CPF3.5). A commercially available product of chicory inulin, with a dry matter of 96%, containing 90% inulin with an average polymerization degree of 10% and a free sugar content of 10% was added to drinking water and the average inulin consumption was 3.73 mg ± 0.04 mg/g of BW/day.

### Animals and treatment

#### Dams

All procedures were carried out according to the Animal Care and Use Committee at Jules Verne University of Picardy (n°291112–19, Amiens, France) which approved this study. All efforts were made to minimize animal’s suffering. Wistar rats, thirty-two females and five males (Janvier, Le Genest St Isle, France) were housed in breeding cages under constant conditions of ambient temperature (23°C), hygrometry (26%), with a 12h light/dark cycle and free access to food and water. After 1 week of acclimation period, females were mated with males (2 females per male). Time-pregnant, primiparous Wistar rats were determined by the presence of spermatozoa in vaginal smear. Pregnant females were individually housed and randomly assigned (1:1) to five treatment groups (n = 5 to 6) and a control group (n = 5). In each treatment group, the dams, from the first gestation day (GD) until lactation day (LD) 21, were exposed to a daily gavage of vehicle or CPF associated with or without inulin. The different treatment groups were as follows: CPF groups were CPF0, CPF1 and CPF3.5 and inulin groups were inu0 and inu1. CPF amounts were adjusted daily according to any changes in the body weight and administered at approximately the same time each morning. Each pregnant female’s food and drink intake were recorded every three days from GD 0 to LD 21.

#### Pups

At postnatal day 1 (PND 1), all pups were counted, sexed and weighed. Each litter was homogenized and adjusted to 8 pups. At PND 21, only male pups were weaned and received the same treatment as dams. Birth weight, food and drink intake were recorded every 2 days from PND 1 to PND 60. At PND 60, animals were euthanized by intraperintoneal administration of lethal dose of sodium pentobarbital (Ceva Santé Animale (Libourne, France) and a sample of blood, brain, liver, fecal content and colon were collected. Tissues were immediately frozen and stored at -80°C. Blood samples were centrifuged at 4000g for 10min and plasma was collected, aliquoted and stored at -80°C.

### Metabolic assay

Plasma leptin and insulin levels were measured by specific commercial RIA kit (EMD, Millipore, France). Standard spectrophotometric methods based on an automation program by Amiens University Hospital were used for measurement of the following serum parameters: Cholesterol, High Density Lipoprotein (HDL), Low Density Lipoprotein (LDL), triglycerides (TG), glycaemia, alanine transaminase (ALT) and aspartate transaminase (AST).

### Protein extraction from liver

Proteins were extracted from 20 mg of liver tissue according to methods described in our previous studies [26,27]. Briefly, tissues were lysed in 1X Ripa Buffer containing a cocktail of protease inhibitors (Thermo Scientific, France). Lysis was performed using a Tissue Lyser device (Qiagen, France). Samples were incubated for 15–20 min at 4°C and centrifuged (14000g; 15 min; 4°C). The supernatant was removed and stored at -80°C. Protein concentrations were measured using a Pierce BCA Protein Assay kit according to the manufacturer’s instructions.

### Gel electrophoresis and Western Blot analysis

For gel electrophoresis, the protein samples were resuspended and heated for 5 min at 95°C and loaded on a 4–12% SDS-PAGE (Criterion XT Bis-Tris Gel, Biorad, France). Proteins were transferred onto a nitrocellulose membrane (Biorad, Fance) after electrophoresis. Membranes were blocked with TBS-T/5% milk and incubated overnight at 4°C with the primary antibody anti insulin receptor β (IRβ) (rabbit monoclonal, #3020, Cell Signaling, diluted 1/1000). The blot was then incubated with peroxidase conjugated secondary antibody (Abcam, diluted 1/5000). The protein signal was detected using the ECL kit (Amersham Biosciences). Proteins were analyzed using anti- Signals on autoradiographic films were quantified by scanning densitometry using ImagQuant 350 (GE Healthcare, France).

### DNA Extraction and 16S RNA qPCR analysis

Total bacterial count and specific bacterial profile were evaluated by quantitative PCR analyses targeting bacterial group-specific 16S rRNA genes using the Rotor-Gene system (Qiagen, France). Total DNA was extracted from 20 to 25 mg of colon content using the Qiagen QIAamp Fast DNA stool kit according to the manufacturer’s instructions (Qiagen, France). PCR inhibitions were tested with TaqMan® Exogenous Internal Positive Control and the TaqMan® universal master Mix (Life Technologies S.A, France). No PCR inhibition was detected using 10^−3^ dilutions for the feces samples. DNA from each sample was amplified using selected primers and probe sets given in Table 1 [28,29]. For bacterial SYBR-green amplification studies, a melting curve was added to show the amplification specificity and the following PCR profile was used: 1 cycle at 95°C for 12 min, followed by 40 cycles of 95°C for 15 s, 60°C for 30 s, 72°C for 30 s. Cycle amplification data were quantified according to standard curves. Data were expressed as log10 (copy number)/g of feces.

**Table 1:** Primers and probes used in this study.

	Traget	Primers and Probes	Sequences (5’-3’)
TaqMan System	All bacteria^a^	F-Bact 1369	CGGTGAATACGTTCCCGG
		R-Prok 1492	TACGGCTACCTTGTTACGACTT
		**P-TM1389F**	**6FAMCTTGTACACACCGCCCGTC**
	*Bifidobacterium*^a^	F-Bifid 09c	CGGGTGAGTAATGCGTGACC
		R-Bifid 06	TGATAGGACGCGACCCCA
		**P-Bifid**	**6 FAM CTCCTGGAAACGGGTG**
	*Clostridium leptum* group^a^	F-Clept09	CCTTCCGTGCCGSAGTTA
		R-Clept 08	GAATTAAACCACATACTCCACTGCTT
		**P-Clep 01**	**6 FAM-CACAATAAGTAATCCACC**
	*Clostridium coccoides* group^a^	F-Ccoc07	GACGCCGCGTGAAGGA
		R-Ccoc14	AGCCCCAGCCTTTCACATC
		**P- Erec482**	**VIC-CGGTACCTGACTAAGAAG**
SyberGreen System	*Bacteroidetes*^b^	BactF	CCTWCGATGGATAGGGGTT
		BactR	TCCCCAGGTGGAATACTTAACG
	*Lactobacillus/Leuconostoc/Pediococcus*^a^	F-lacto05	AGCAGTAGGGAATCTTCC
		R-lacto04	CGCCACTGGTGTTCYTCCATATA
	*Firmicutes*^b^	FirmF	ACCCGCGTCTGATTAGCTAGTT
		FirmR	CCTCTCAGGCCGGCTACTG

^a^ [28]

^b^ [29]

### Short Chain Fatty Acid (SCFA) analysis

The concentration of acetate, propionate and butyrate in the fecal material were determined after water extraction of acidified samples using gas-liquid chromatography (Thermo Scientific, Focus GC-AutoInjector AI 3000) as described in our previous study [30].

### Statistical analysis

Statistical analyses were performed with StatView software (version 5.0, Abacus Concepts Inc., Berkeley, CA, USA). The non-parametric tests Kruskal Wallis was used to analyze the effects of CPF ([CPF0, CPF1, CPF3.5] inu0, [CPF0, CPF1, CPF3.5] inu1) followed by Mann Whitney test, when significance reached *p*<0.05. Effects of inulin was analyzed by Mann Whitney test ([inu0,inu1]CPF0, [inu0,inu1]CPF1, [inu0,inu1]CPF3.5). All results were presented as means ± standard error of the mean (SEM). Significance was set at the value of *p*<0.05 and the indicative results were presented if relevant. Due to birth body weight variation among the different groups, body weight change from birth to PND60 was adjusted accordingly to birth body weight (BW PND1): ((PND60-BW PND1)/BW PND1).

## Results

### Dam’s body weight, food and drink intake (GD 0 and LD 21)

Dam’s body weight, food intake and drink intake were measured every 3 days. Neither CPF nor inulin significantly affected maternal weight gain, food intake and drink intake (S1 Table). No signs of cholinergic toxicity such as tremor, salivation or diarrhea were observed in animals during the whole experiment.

### Offspring’s body weight and growth, food and drink intake

**Fig 1:**
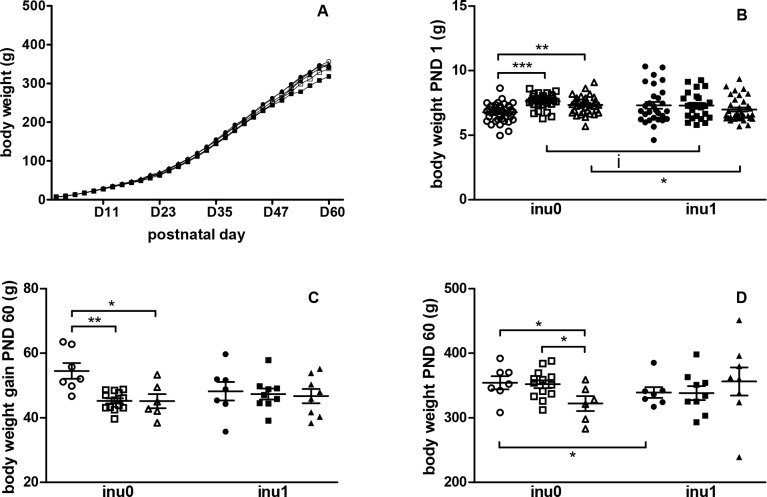
Offspring’s body weight and growth. (A) Global growth of animals from PND1 to PND 60; (B) Body Weight at PND 1 [CPF0inu0 n = 38/CPF1inu0 n = 31/CPF3.5 n = 35/CPF0inu1 n = 29/CPF1inu1 n = 25/CPF3.5inu1 n = 34]; (C) BW gain PND 60; (D) BW at PND 60 of male pups. The results are expressed as mean ± SEM and individual values and analyzed by Mann Withney test. Control groups (CPF0, circles), CPF-exposed groups (CPF1: 1 mg/kg/day, squares; CPF3.5: CPF3.5 mg/kg/day, triangles) or inulin groups (black symbol). *Signification* *p<0.05; **p<0.01,***p<0.001, i = 0.058.

Offspring’s body weight, growth and food intake were recorder every two days from PND1 to PND60. The global growth curve of different groups did not appear to be affected by treatments except CPF3.5 animals. Indeed, a “drop” of the curve for these exposed rats to the highest dose of CPF was observed at PND53 (Fig 1A). The mean BW of control offspring group at birth and PND 60 were 6.77 ± 0.12 g and 355.9 ± 8.2 g, respectively. Inulin alone did not affect offspring BW and BW gain but animal’s weight at PND 60 decreased with inulin supplementation (p<0.05) (Fig 1D). Gestational exposure to CPF significantly increased BW at birth (p<0.001) (Fig 1B). BW gain and BW at PND 60 were decreased in both CPF-exposed groups (p<0.05) (Fig 1C and 1D). This reduction in weight gain was not associated with changes in either food or water intake (S2 Table). The supplementation of inulin in CPF-exposed groups induced a decrease of BW at birth as compared with CPF animals. However, no effects of inulin on BW gain and BW at PND 60 were observed in CPF-exposed animals (Fig 1C and 1D). Thus CPF exposure modified the body weight growth pattern of the offspring’s from birth to weaning. In both CPF exposed rats, inulin supply induced a decrease of birth body weight and restored the BW at PND 60 to control levels.

### Metabolic parameters

**Fig 2:**
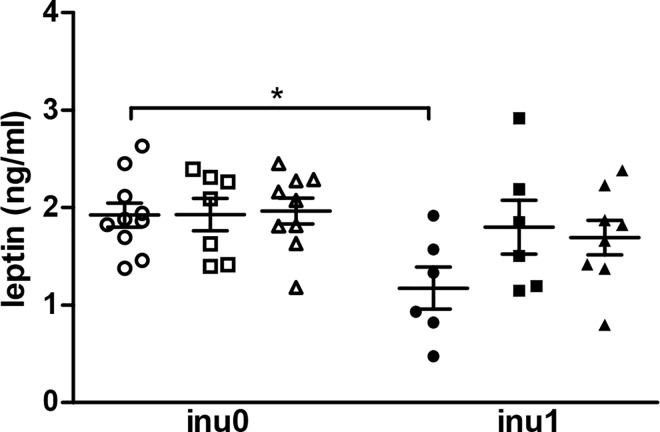
Leptin level of male pups at PND 60. Data are expressed as mean ± SEM [CPF0inu0 n = 10/CPF1inu0 n = 7/CPF3.5 n = 9/CPF0inu1 n = 6/CPF1inu1 n = 6/CPF3.5inu1 n = 8]. Control groups (CPF0, circles), CPF-exposed groups (CPF1: 1 mg/kg/day, squares; CPF3.5: CPF3.5 mg/kg/day, triangles) or inulin groups (black symbol). *Signification* *p<0.05; **p<0.01,***p<0.001.

**Fig 3:**
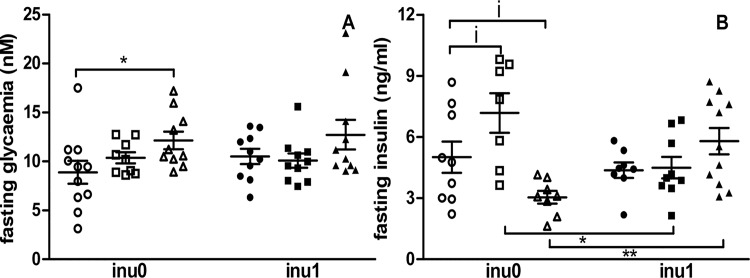
Fasting Glycaemia and Insulin profile. (A) Fasting Glycaemia [CPF0inu0 n = 11/CPF1inu0 n = 9/CPF3.5 n = 10/CPF0inu1 n = 10/CPF1inu1 n = 10/CPF3.5inu1 n = 10]; (B) Fasting Insulin are expressed as mean ± SEM [CPF0inu0 n = 9/CPF1inu0 n = 7/CPF3.5 n = 8/CPF0inu1 n = 8/CPF1inu1 n = 9/CPF3.5inu1 n = 11]. Control groups (CPF0, circles), CPF-exposed groups (CPF1: 1 mg/kg/day, squares; CPF3.5: CPF3.5 mg/kg/day, triangles) or inulin groups (black symbol). *Signification* *p<0.05; **p<0.01,***p<0.001, i = 0.08.

The effect of the different treatments on metabolic parameters was compared between the different groups. Inulin alone did not affect metabolic parameters while leptin level at PND 60 decreased when inulin was supplied in the drinking water (p<0.05) (Fig 2). CPF exposure during pregnancy and lactation induced different effects on the metabolic parameters depending on the dose used: CPF3.5 group showed a significant higher increase in glycaemia at PND 60 (p<0.05) compared to the control (Fig 3A). A slight increase in plasma insulin level (p = 0.08) at PND 60 was observed in CPF1-exposed offspring’s while a slight decrease in insulin secretion was observed in animals exposed to the higher dose (p = 0.08). When supplemented with inulin, the levels of insulin in both CPF1 and CPF3.5 groups were close to the values observed in control animals (p<0.05) (Fig 3B).

**Fig 4:**
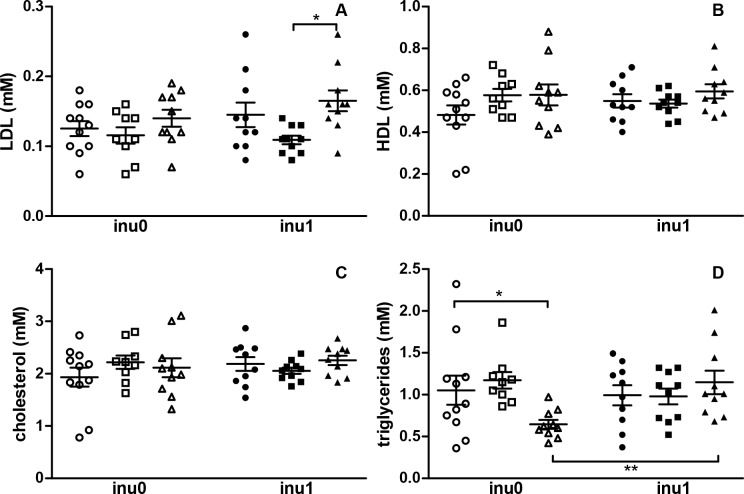
Plasma lipids status. (A) LDL; (B) HDL; (C) cholesterol; (D) triglycerides are expressed as mean ± SEM [CPF0inu0 n = 11/CPF1inu0 n = 9/CPF3.5 n = 10/CPF0inu1 n = 10/CPF1inu1 n = 10/CPF3.5inu1 n = 10]. Control groups (CPF0, circles), CPF-exposed groups (CPF1: 1 mg/kg/day, squares; CPF3.5: CPF3.5 mg/kg/day, triangles) or inulin groups (black symbol). *Signification* *p<0.05; **p<0.01,***p<0.001.

**Fig 5:**
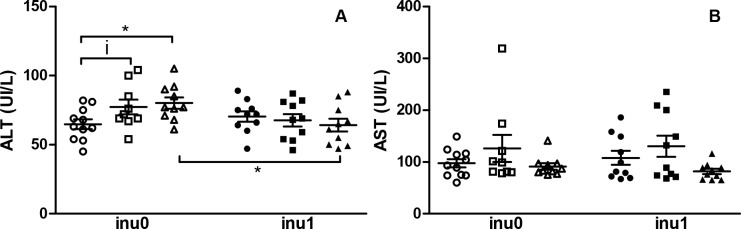
Plasma liver enzymes profile. (A) ALT; (B) AST are expressed as mean ± SEM [CPF0inu0 n = 11/CPF1inu0 n = 9/CPF3.5 n = 10/CPF0inu1 n = 10/CPF1inu1 n = 10/CPF3.5inu1 n = 10]. Control groups (CPF0, circles), CPF-exposed groups (CPF1: 1 mg/kg/day, squares; CPF3.5: CPF3.5 mg/kg/day, triangles) or inulin groups (black symbol). *Signification* *p<0.05; **p<0.01,***p<0.001, i = 0.07.

The lipid status (i.e., the total cholesterol, LDL and HDL) was not affected by inulin or CPF exposure alone except for the level of TG which was lower in CPF3.5-exposed rats as compared to control animals (p<0.05). The TG level was recovered when animals were co-exposed with inulin and CPF3.5 in comparison to CPF3.5 animals alone (p<0.01) (Fig 4). Fig 5A shows an increase in ALT enzyme for the CPF3.5-exposed groups (p<0.05) and a slight increase (p = 0.07) in ALT in CPF1 exposed animals compared to the control. The difference in ALT level did not persist when animals received both CPF3.5 and inulin. Meanwhile, no changes in AST level were observed with the different treatments (Fig 5B). Thus CPF modified the metabolic status of the offspring’s and inulin restored the levels of some metabolic parameters in CPF groups to ranges comparable with the controls.

### Insulin protein expression

**Fig 6:**
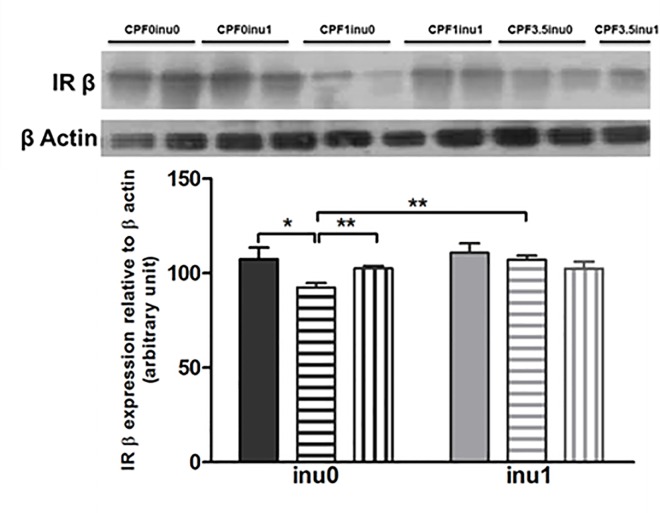
Determination of IRβ in liver of rats at PND 60 by Western blot. Data are expressed as mean ± SEM [CPF0inu0 n = 5/CPF1inu0 n = 5/CPF3.5 n = 5/CPF0inu1 n = 5/CPF1inu1 n = 4/CPF3.5inu1 n = 4]. Control groups (CPF0, circles), CPF-exposed groups (CPF1: 1 mg/kg/day, squares; CPF3.5: CPF3.5 mg/kg/day, triangles) or inulin groups (black symbol). (n = 8 to 10). *Signification* *p<0.05; **p<0.01,***p<0.001.

Western blot analysis evidenced a significant (p<0.05) decrease of IRβ protein expression in the liver of CPF1 as compared to controls. When supplemented with inulin, IRβ was significantly (p<0.01) recovered only in CPF1-exposed group (Fig 6A and 6B).

### Gut microbiota

**Fig 7:**
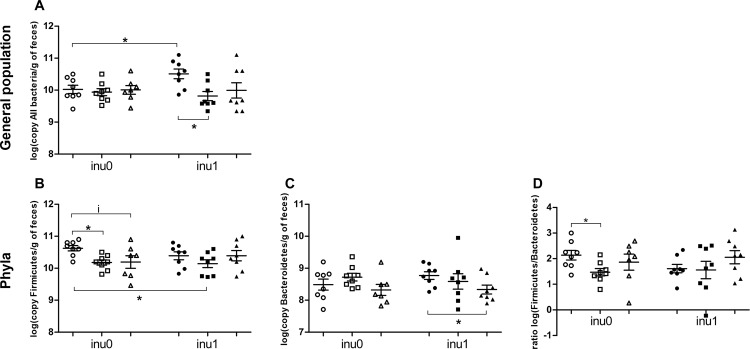
General bacterial profile. (A) All Bacteria; (B) *Firmicutes*; (C) *Bacteroidetes*; (D) *Firmicutes/Bacteroidetes* ratio; are expressed as log(number of copy/g of feces) mean ± SEM [CPF0inu0 n = 8/CPF1inu0 n = 8/CPF3.5 n = 7/CPF0inu1 n = 8/CPF1inu1 n = 8/CPF3.5inu1 n = 8]. Control groups (CPF0, circles), CPF-exposed groups (CPF1: 1 mg/kg/day, squares; CPF3.5: CPF3.5 mg/kg/day, triangles) or inulin groups (black symbol). *Signification* *p<0.05; **p<0.01,***p<0.001, i = 0.09.

**Fig 8:**
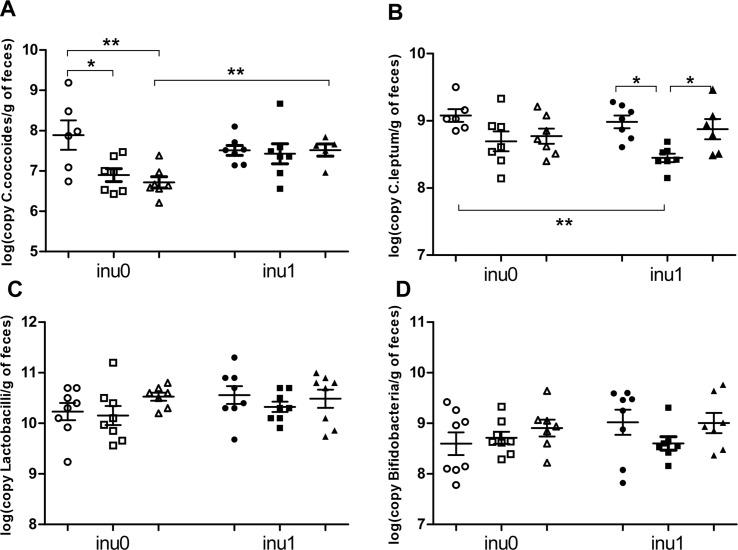
Bacterial genus profile. (A) *C*. *coccoides* group; (B) *C*. *leptum* group; (C) Lactobacilli; (D) Bifidobacteria are expressed as log(number of copy/g of feces) mean ± SEM [CPF0inu0 n = 6-8/CPF1inu0 n = 7-8/CPF3.5 n = 7/CPF0inu1 n = 7-8/CPF1inu1 n = 7-8/CPF3.5inu1 n = 5–8]. Control groups (CPF0, circles), CPF-exposed groups (CPF1: 1 mg/kg/day, squares; CPF3.5: CPF3.5 mg/kg/day, triangles) or inulin groups (black symbol). *Signification* *p<0.05; **p<0.01,***p<0.001.

Total bacterial count and specific bacterial profile were evaluated by quantitative PCR analyses targeting bacterial group-specific 16S rRNA. Perinatal exposure to inulin alone significantly increased the global bacterial population (p<0.05) (Fig 7A) and total SCFA production (p<0.01) with a significant increase in the proportion of butyric acid (p<0.05) (Table 2). A significant decrease in the population of *Firmicutes* (Fig 7B) was observed in CPF-exposed animals (p<0.05) for CPF1 and a slight decrease (p = 0.08) for CPF3.5 respectively. Perinatal exposure to CPF alone significantly decreased *C*. *coccoides* group (p<0.05) and tended to reduce *C*. *leptum* count too (Fig 8A and 8B). In the CPF3.5+inu group, C. *coccoides* group was significantly (p<0.01) higher than CPF3.5-exposed rats (Fig 8A). The *Firmicutes/Bacteroidetes* ratio decreased only in the CPF1 group in comparison to the control (p<0.05) (Fig 7D). In the CPF1+inu group, the total count of *Firmicutes* population and *C*. *leptum* group were still significantly lower when compared to the control group (p<0.05) (Figs 7B and 8B). For the production of SCFAs, supplementation of CPF1 animals with inulin increased the total SCFAs production (p<0.05) as well as the proportion of butyric acid (p = 0.058) compared to CPF1 animals without inulin. However, in CPF3.5 group, inulin supplementation only increased the proportion of butyric acid (p = 0.058) when compared to CPF3.5 without inulin. Of note, a high decrease of total SCFAs was observed in CPF3.5 animals supplemented with inulin as compared to the group receiving only inulin (305 ± 16 μmol/g *vs*. 393 ± 27,1 μmol/g respectively, p<0.01). Thus, CPF decreased *Firmicutes* population. Inulin supplementation enhanced the total bacterial population, SCFA production and butyrate levels in CPF groups.

**Table 2:** Bacteria fermentation activity in offspring at PND 60.

	CPF0		CPF1		CPF3.5	
	inu0	inu1	inu0	inu1	inu0	inu1
SCFA (μmol/g)	289.5±15.3	393.0±27.1**^a^	263.1±14.3	314.0±14.4*^b^*^d^	282.1±18.4	305.0±16.0**^e^
Acetic acid (%)	75.3±0.55	73.4±1.01	75.0±0.55	74.23±1.28	75.2±0.46	73.7±0.75
Propionic acid (%)	16.4±0.74	15.9±0.70	17.3±0.63	17.0±0.94	15.3±0.55	15.0±0.71
Butyric acid (%)	8.25±0.44	10.73±0.56*^a^	7.80±0.29	8.67±0.70 i^b^*^d^	9.56±0.30	11.28±0.67i^c^

Data are expressed as mean ± SEM. Groups: CPF0inu0 n = 14, CPF0inu1 n = 12, CPF1inu0 n = 15, CPF1inu1 n = 11, CPF3.5inu0 n = 13, CPF3.5inu1 n = 14.

^a^ CPF0inu0 vs. CPF0inu1

^b^ CPF1inu0 vs. CPF1inu1

^c^ CPF3.5inu0 vs. CPF3.5inu1

^d^ CPF0inu1 vs. CPF1inu1

^e^ CPF0inu1 vs. CPF3.5inu1

Signification

*p<0.05

** *p*<0.01; i = 0.058

## Discussion

This study demonstrates that CPF exposure during a critical window of development induced permanent defects in normal newborn male rats in terms of their phenotype and their metabolic regulation during adulthood. The exposure to CPF during gestation led to an increase in BW when the offspring were born. However, a long term exposure extending from gestation to adulthood induced a decrease in BW gain and BW in the offspring at day 60. Perturbations in the perinatal environment can impair metabolic programming that can consequently increase susceptibility to T2D in adulthood. Several studies addressing the role of developmental exposure to pesticides with diabetes have rapidly expanded over the past years suggesting that they contribute to metabolic programming [9,31]. In our study we demonstrate that a continuous exposure to a “safe” dose of CPF beginning in the womb until adult life impairs adult metabolism of the offspring’s leading to insulin and lipid dysregulation. Based on these findings, one can assume that the exposure to CPF during gestation, lactation and even during other sensitive periods of development, as pre-puberty, impairs developmental programming. Although our design does not able determining precisely which of the different targets sensitive windows were involved in the “programmed” metabolic defects, it complements the Barker hypothesis which makes a link between low birth weight and diabetes [32], extending the same outcomes into the situation to developmental exposure to pesticides, even in the absence of growth retardation. The specific pattern of birth weight increase and BW loss after pre- and postnatal exposure to CPF differs from studies published by others on exposure to CPF [33] or Parathion [34], which is another organophosphorous in neonates. This discrepancy can be explained by the different doses used, the time window, the type (acute or chronic) of exposure and the mode of administration (oral, subcutaneous) in different studies.

The major drastic effects of CPF exposure on glucose and lipid metabolism seen in the 60 days old offspring rats were observed in those subjected to the higher dose of 3.5 mg/kg/day CPF. Indeed, an increase in glycaemia concomitant with a drastic reduction of insulin level was observed. This result may reflect an alteration of the pancreas integrity and functionality and as a consequence, the inability of the pancreas to control glycaemia in CPF-exposed rats. Moreover, the alteration of glucose metabolism observed for the higher dose of CPF resulted in a significant increase in serum alanine aminotransferase reflecting hepatic injuries. Indeed, Mansour et al., showed that CPF caused oxidative damage leading to impaired liver cell membrane permeability and thus the release of hepatic enzymes [35]. Furthermore, in association with the decrease in insulin secretion, a subsequent decrease in triglycerides was seen in CPF3.5-exposed rats. As insulin is an important regulatory factor of lipid metabolism [36], one can assume that an alteration in insulin secretion perhaps modified the triglyceride content. Other mechanisms can also contribute to the disturbance of triglyceride synthesis, notably the ones targeting adipose tissue and/or liver signaling that are essential for homeostasis regulation [37,38]. We recently reported that CPF exposure increased para-cellular permeability in the small intestine [39] and delayed intestinal epithelial maturation [25], which may suggest a modification of intestinal absorption of triglycerides in the blood stream.

In agreement with the studies by Slotkin [9,10], CPF1-exposed rats presented a hyperinsulinemia. This result showed that an apparently “safe” exposure was probably maintained in the CPF1-group by the compensatory hypersecretion of insulin. This situation is close to what was observed in a pre-diabetic state. We also reported that the liver of CPF1 animals contain reduced amounts of IRSβ, which support our hypothesis and reflect a compensatory response to chronically elevated insulin level and alteration of insulin sensitivity [40].These findings highlighted a mechanistic link between insulin signaling in the liver and the subsequent emergence of hyperinsulinemia and hyperlipidemia.

The original approach used in this study was based on the use of inulin, acting as a prebiotic to counteract the side effects of CPF on metabolism. Prebiotics are known to improve the host's health by inducing favorable changes in intestinal microbiota [41]. Inulin selectively stimulates beneficial Bifidobacteria and Lactobacilli *in vitro* [42,43] and also in human subjects and rodents [44–46].

Intestinal SCFA concentration, especially butyrate, the preferred energy source of colonocytes, increases when inulin is consumed [45,46]. In agreement with this, inulin supplementation resulted in an increase in the general population of bacteria and the total SCFA production. An increase in SCFA, observed in supplemented groups, may explain the reduction of insulin level noticed in CPF1-exposed rats because of the role of SCFAs in the activation of G-protein–coupled free fatty acid receptor (GPR43) in the adipose tissue [47]. Moreover, the observed increase in butyrate in rats consuming inulin is probably due to a cross-feeding phenomenon such that the butyrate-producing bacteria belonging to *C*. *coccoides* (cluster XIVa) and *C*. *leptum* group (cluster IV) such as *Roseburia intestinalis* or *Faecalibacterium prausnitzii* or others clostridial clusters such as I, III, XI, XV, XVI [48] which use acetate produced by Bifidobacteria [49]. To our knowledge, we are the first to describe a decrease in *Firmicutes*, *C*. *coccoides* and *C*. *leptum* group in particular, after a perinatal CPF exposure in rats. This microbiota pattern resembles what we observed in the Human diabetic subjects with a decrease of *Firmicutes* and particularly butyrate-producing Clostridiales [50,51], *Bacteroides vulgates*, and Bifidobacteria [52]. It is well known that aberrant intestinal microbiota can induce a translocation of bacterial fragments and the development of “metabolic endotoxemia”, leading to systemic inflammation and insulin resistance [17]. Access to inulin may counteract endotoxemia in CPF-exposed rats as it alleviates the decrease in the phylum *Firmicutes* and clostridial clusters XIVa (*C*. *coccoides*) and IV (*C*. *leptum*).

In addition to the beneficial effect of inulin in CPF-exposed rats on microbiota and SCFA production, our results showed that it modified the metabolic status of the CPF-exposed rats according to the dose used. In CPF3.5 group, free access to inulin induced an increase in insulin, triglycerides and alanine transferase in the serum to a level close to those of control groups, showing that inulin restored some features of the disturbed metabolic profile in CPF3.5-exposed animals. Conversely, in CPF1-exposed rats, inulin decreased insulin secretion, initially high in CPF1-exposed rats, to a level comparable to those of control animals. Moreover, prebiotics increased the expression of IRβ in CPF1-exposed rats suggesting an enhancement of insulin sensitivity and a decrease of insulin resistance in these animals. Thus, altogether, our results show that our study is the first to demonstrate that bringing a continuous source of prebiotic to the mother and the infant, allowed young rats to alleviate the side effect of long-term exposure to CPF on their metabolism during their adult lives.

## Conclusions

In summary, the current results indicate that CPF exposure during pre- and postnatal period may be a limiting factor, at least in rodents, for the onset of a normal regulation of metabolism in the offspring during their adult life. It is suggested that organophosphate insecticides can increase the risk of diabetes mellitus. We propose that prebiotics, which have the ability to alter the microbiota in a positive manner, is a safe and cost-effective nutritional strategy to counteract CPF insulin resistance and diabetes in later life. Human clinical trials should be undertaken to confirm these effects. However, additional basic research is necessary to better understand the crosstalk between microbiota and the host in order to elucidate the exact mechanism by which, microbiota alleviates the metabolic defects induced by CPF in adults. Experiments are in progress to determine whether the gut/brain axis is also involved in such regulation.

## Supporting Information

S1 Table Dam’s body weight, food and drinking intake during gestation and lactation periods. Data are expressed as mean ± SEM and analyzed by Mann Withney test. Groups: CPF0inu0, CPF0inu1, CPF1inu0, CPF1inu1, CPF3.5inu0, CPF3.5inu1.
(DOCX)

S2 Table Pup’s food and drinking intake from PND 21 to PND 60. Data are expressed as mean ± SEM and analyzed by Mann Withney test. Groups: CPF0inu0, CPF0inu1, CPF1inu0, CPF1inu1, CPF3.5inu0, CPF3.5inu1.
(DOCX)
