# Supplementary material for: Inulin Supplementation Lowered the Metabolic Defects of Prolonged Exposure to Chlorpyrifos from Gestation to Young Adult Stage in Offspring Rats
Source: PLoS One. 2016 Oct 19;11(10):e0164614. doi: 10.1371/journal.pone.0164614 (PMC5070743; doi:10.1371/journal.pone.0164614)
Supplement: S1 Table — Data are expressed as mean ± SEM and analyzed by Mann Withney test. Groups: CPF0inu0, CPF0inu1, CPF1inu0, CPF1inu1, CPF3.5inu0, CPF3.5inu1. (DOCX) [file pone.0164614.s001.docx]

|  | CPF0 | | CPF1 | | CPF3.5 | |
| --- | --- | --- | --- | --- | --- | --- |
|  | inu0 | inu1 | inu0 | inu1 | inu0 | inu1 |
| *body weight gain (g)* |  |  |  |  |  |  |
| gestation | 0.24±0.03 | 0.24±0.03 | 0.23±0.05 | 0.24±0.03 | 0.22±0.03 | 0.24±0.03 |
| lactation | 0.11±0.03 | 0.08±0.03 | 0.09±0.02 | 0.08±0.02 | 0.07±0.03 | 0.11±0.04 |
| *food intake (g/g of BW*) |  |  |  |  |  |  |
| gestation | 0.24±0.01 | 0.24±0.01 | 0.24±0.01 | 0.25±0.02 | 0.23±0.01 | 0.23±0.02 |
| lactation | 0.55±0.04 | 0.48±0.05 | 0.58±0.09 | 0.48±0.06 | 0.54±0.04 | 0.52±0.03 |
| *drinking intake (ml/g of BW)* |  |  |  |  |  |  |
| gestation | 0.41±0.02 | 0.45±0.07 | 0.38±0.07 | 0.38±0.03 | 0.30±0.06 | 0.37±0.07 |
| lactation | 0.82±0.08 | 0.69±0.10 | 0.69±0.09 | 0.71±0.06 | 0.70±0.10 | 0.70±0.07 |
