# Supplementary material for: Inulin Supplementation Lowered the Metabolic Defects of Prolonged Exposure to Chlorpyrifos from Gestation to Young Adult Stage in Offspring Rats
Source: PLoS One. 2016 Oct 19;11(10):e0164614. doi: 10.1371/journal.pone.0164614 (PMC5070743; doi:10.1371/journal.pone.0164614)
Supplement: S2 Table — Data are expressed as mean ± SEM and analyzed by Mann Withney test. Groups: CPF0inu0, CPF0inu1, CPF1inu0, CPF1inu1, CPF3.5inu0, CPF3.5inu1. (DOCX) [file pone.0164614.s002.docx]

|  | CPF0 | | CPF1 | | CPF3.5 | |
| --- | --- | --- | --- | --- | --- | --- |
|  | inu0 | inu1 | inu0 | inu1 | inu0 | inu1 |
| *food intake (g/g of BW*) | 0.27±0.01 | 0.27±0.01 | 0.28±0.01 | 0.27±0.01 | 0.28±0.01 | 0.28±0.01 |
| *drinking intake (ml/g of BW)* | 0.35±0.01 | 0.38±0.01 | 0.37±0.02 | 0.35±0.01 | 0.38±0.01 | 0.40±0.01 |
